# Psychotic Symptoms and Malignant Neuroleptic Syndrome in Williams Syndrome: A Case Report

**DOI:** 10.3389/fpsyt.2022.891757

**Published:** 2022-05-31

**Authors:** Boris Karpov, Maria Muhonen, Tuula Kieseppä

**Affiliations:** ^1^Department of Psychiatry, HUS Helsinki University Hospital, Helsinki, Finland; ^2^Ministry of Social Affairs and Health, Helsinki, Finland

**Keywords:** psychosis, malignant neuroleptic syndrome, Williams syndrome, COVID-19, psychotic symptom

## Abstract

**Background:**

Somatic and mental comorbidities are characteristic of individuals with Williams syndrome. The psychiatric profile of these patients mainly comprises affective disorders, while psychotic symptoms are rare.

**Methods:**

We present a case report of psychosis and malignant neuroleptic syndrome in a patient with Williams syndrome. We also conduct a review of recent works on the topic.

**Case Presentation:**

A 38-year-old Caucasian male with Williams syndrome presented with somatic delusions, previously experiencing severe anxiety and concerns about a headache. The patient was prescribed olanzapine, which did not, however, have any effect on the delusions. After switching to lurasidone, the patient presented with malignant neuroleptic syndrome (muscle rigidity, tremor, urinary retention, fluctuating level of consciousness). He was hospitalized and the antipsychotic medication was discontinued. After somatic recovery, the patient did not experience severe anxiety and the somatic delusions diminished notably. The patient was discharged from the hospital in a stable physical condition, albeit still with transient worries about his health condition.

**Conclusions:**

We present a case of the coincidence of Williams syndrome and psychosis. We hypothesize on the possible pathological relationships between the onset of the psychosis and severe anxiety in an individual with Williams syndrome. This case report duly contributes to the limited literature on psychiatric comorbidity in Williams syndrome.

## Background

Williams Syndrome (WS) is a rare genetic disorder caused by chromosome 7q11.23 contiguous gene deletion (1). The disorder is characterized by a distinctive facial appearance and growth abnormalities. Clinical manifestation of WS often includes cardiovascular diseases and endocrine dysregulations, such as hypercalcemia, hypothyroidism and a predisposition to diabetes (1, 2). Aside from somatic features, WS is associated with neurocognitive abnormalities, such as delays in cognitive development and weaknesses in visuospatial and number cognition (3). Moreover, many individuals with WS have a complex behavioral construct that combines high levels of empathy and hypersociality with emotional difficulties and anxiety (3–5). Anxiety, both trait and state, appears to be the most common psychiatric comorbidity in individuals with WS, followed by attention-deficit/hyperactivity disorder and depression (3, 6, 7). On the contrary, only a limited number of cases of psychotic comorbidity in WS appear in the literature. Although some authors report rates of psychotic disorders in WS as 2–5% (6, 8, 9), the actual number of patients with psychosis in these studies remains very low, usually not exceeding single individuals. In comparison, in patients with more common 22q11.2 Deletion Syndrome lifetime prevalence of psychotic disorders is estimated at 32% (10), while lifetime prevalence of psychotic disorders in general population varies between 2.5 and 3.5% (11, 12).

A recent report by Valdes et al. (7) presents five cases (three original cases and two literature overviews) of WS accompanied by mood disorder with psychotic features. However, we identified only two case reports on WS with psychotic symptoms beyond a mood component (13, 14), considering such a condition extremely rare.

Recent study of Thom et al. (15) review available evidence on the use of psychiatric medications in WS. However, data on use of antipsychotic medications are limited to the case reports mentioned above (13, 14). Thus, no conclusions could be made so far on a safety and tolerance profile of antipsychotic for the patients with WS.

Below, we present the medical history and treatment strategy of a patient with WS and an acute psychotic episode.

## Case Presentation

A 38-year-old Caucasian male with Williams syndrome (established at the age of three) had suffered from a severe headache, which interfered with his sleep and daily functioning. Six months prior to the manifestation of the headache, the patient was prescribed candesartan 4 mg for hypertension. After 2 weeks of suffering with the headache, the patient sought emergency care. Physical and laboratory examinations did not reveal any signs of serious somatic or neurological disease; the results of a computed tomography scan were normal. The patient was diagnosed with tension headache and was provided with proper self-treatment instructions and medication (paracetamol, ibuprofen). Soon after his first visit to emergency care, the patient reported feeling a “trembling blood vessel” in his head. During the following 2 months, the patient experienced only slight relief from the headache and complained of a “pulsing” feeling in his head. Additional magnetic resonance imaging did not reveal any pathological changes in the brain structures. More recently, the patient had complained of tinnitus and “hearing his own pulse” in his ear. Trials of betahistine and tizanidine were provided with no effect. The patient was highly concerned about the cause of the headache and “pulsation feeling” and became anxious to an increasing extent. Eventually, the patient presented with an intense delusion of having a worm in his brain. As a consequence, the patient suffered from severe anxiety and psychomotor agitation. With the assistance of his mother, the patient sought emergency psychiatric care. He was prescribed olanzapine 5 mg two times a day and oxazepam 7.5–15 mg 1–3 times a day. During the clinical examination, the patient was clearly anxious, although no signs of depression emerged. Based on the clinical picture, the patient was referred to the First-episode Psychosis Outpatient Clinic.

In addition to neuroimaging tests, the patient underwent an exhaustive laboratory examination during the initiation of psychiatric treatment. A full blood count, electrolytes and C-reactive protein showed normal values. Tests also showed normal levels of plasma calcium and albumin. No abnormalities emerged in the liver, renal (creatinine and glomerular filtration rate), thyroid (thyroid-stimulating hormone and free thyroxine) and parathyroid functions. During olanzapine monotherapy, the drug's serum level remained within the therapeutic interval. The only abnormality found in the laboratory tests was hyperprolactinemia (serum prolactin 695 mU/l), which did not require any interventions according to the endocrinology consultant.

There was no evidence of prior psychiatric illness or symptoms, aside of occasional anxiety, which did not require a medical care, however. There was also no case of mental or neurological disease in his family. Patient was granted a disability pension. Prior to COVID pandemic lockdown, patient attended communal workshop center three times a week and played in a musical band. Patient enjoyed social interactions in workshop center. Patient had his own apartment but spent much time at his parent's house.

During lockdown, workshop center visits and band rehearsals were discontinued. Patient was only able to maintain contact with his parents, not always face-to-face. Patient was frustrated and stressed by social isolation, as, in his own opinion, he had strong need for social contacts.

Initially, the symptoms of anxiety and feeling of “pulsation in the ear” resolved within 2 weeks after initiation of antipsychotic medication. However, the patient still had a somatic delusion about a worm in the brain, or at least certainty about severe brain pathology that needed to be treated. The patient felt that this problem was not being taken seriously and was quite disappointed and anxious about it. One month after the introduction of antipsychotic medication, his anxiety and delusions again increased, and he presented with lassitude and frustration. The dose of olanzapine was increased to 15 mg daily. Overall, olanzapine was used for 6 weeks in therapeutic dose of 10–15 mg, but patient still experienced anxiety and delusions. Thus, response to olanzapine considered as sub-optimal. Dose increase was not conducted to avoid possible olanzapine-related metabolic side-effects. After six weeks of olanzapine-treatment, it was cross-taper switched to lurasidone 74 mg within 2 weeks.

However, 2 weeks after lurasidone initiation, the patient presented with severe muscle rigidity, tremor, elevated blood pressure, urinary retention, a fluctuating level of consciousness, and elevated creatine kinase (CK). Despite the absence of hyperthermia, these symptoms were interpreted as neuroleptic malignant syndrome (NMS). The patient was admitted to the intensive care unit and lurasidone was immediately discontinued. The patient recovered from NMS within 4 weeks, during which time he did not receive any antipsychotic agent and only used trazodone 50 mg for insomnia. Surprisingly, during the “antipsychotic-free” period, the patient did not report any psychotic symptoms, including “worm in the brain” delusions. However, he was extremely worried about his physical condition. After somatic recovery, the patient began to express feelings that something was wrong with his head again. This time, he was not convinced about the worm in his head, but rather reported weird feelings in the parietal area. After four weeks of hospitalization, the patient was discharged in a relatively stable condition. The patient still referred to an aberrant feeling in his head, although this feeling no longer interfered with his affect. For a short period of time, patient has moved to his parents, as he was concerned about his functional level after discharging. However, no signs of functional impairment emerged, and patient was able to rebuild his routine and daily activities within several weeks, as COVID-related restrictions began to loosen.

## Discussion

In the presented case, the patient developed an acute psychotic episode, which was accompanied by severe anxiety. Although the patient had clear morphological and clinical signs related to WS, he had maintained a generally good health condition. Moreover, careful medical, laboratory, and neuroimaging examinations revealed no condition commonly related to the organic etiology of psychosis. Furthermore, based on a psychiatric examination and several weeks of follow-up, no symptoms of depression, mania or personality disorder emerged. Diagnostically, the psychosis was not related to a substance or physiological condition, nor to a mood disorder. In turn, we assume anxiety as a possible contributor to the onset of the psychosis. The patient was very concerned about his health and the cause of a persistent headache, which made him restless and anxious. The relationship between anxiety and psychosis is complex. Anxiety could be a reaction to disturbing and often intimidating psychotic symptoms (16). On the other hand, emotional dysregulation and anxiety symptoms may sometimes precede delusional or hallucinatory experiences, and often trigger a relapse of psychosis (17). In relation to WS, Cherniske et al. (18) regard psychosis as a transient exaggerated response to life stresses and anxiety disorders. The latter mechanism might be valid for our patient as well, as anxiety seemed to accompany the common symptom of a headache and then possibly contribute to its modification to the somatic delusion of brain abnormality *via* emotional dysregulation. Moreover, during recovery from NMS, the patient was focused on his overall condition, rather than certain isolated symptoms. We assume that severe distress associated with a physical condition has dominated the patient's thought processes and functioning, duly obliterating other disturbing experiences such as somatic delusions.

A noteworthy aspect is that the psychosis had its onset during the COVID-19 pandemic and a lockdown caused by local restrictions. Like many others, our patient had to virtually discontinue the vast majority of his social contacts and activities outside the home. Moreover, communicational habits (including outpatient visits) have rapidly switched to online-based activities. Although individuals with WS are characterized by high empathy and hypersociality, they simultaneously experience difficulties in social-communication and reciprocity skills, such as the ability to form and maintain friendships (19, 20). Thus, it is clear that adjustment to the novel social life circumstances was challenging for our patient and might have contributed to his emotional distress and vulnerability to mental health problems. Moreover, growing evidence demonstrates the emergence of psychotic disorders (sometimes to a severe degree) related to COVID-19 lockdowns and quarantine circumstances (21–23).

It is important to outline the presentation of lurasidone-associated NMS in our patient. NMS is generally a rare condition, more commonly associated with the use of typical rather than atypical antipsychotics (24). To date, we identified only one case report on lurasidone-associated NMS (25). Thus, in addition to the rare clinical symptom of psychosis, our patient also presented with a rare reaction to antipsychotic medication. One might speculate that a non-response to olanzapine and an abnormal reaction to lurasidone might be associated with a genetic profile of WS.

Our case has some common features with the case, presented by Salgado et al. (14). Both patients had no significant medical history prior to the onset of the psychosis. Also, both patients experienced notable side-effects from the antipsychotic medication. In addition, in both cases, patients experienced a significant change in their daily routine and social life prior to the onset of the illness. On contrary, patient, described by Salgado et al. presented with not only delusions but also with auditory hallucinations and had some history of behavioral changes and emphasized suspiciousness prior to the onset of psychosis. Based on this comparison, we cannot conclude on any kind of uniformity of manifestation of the psychosis in patients with WS, but the effect of changes in the daily routine and social contacts is noteworthy.

Few limitations of this case report should be mentioned. During the acute phase of the psychosis, no evaluation of patient's cognitive and functional performance was performed, neither reliable data on evaluation prior to the onset of the illness was available. In addition, no information on individual pharmacokinetic profile was collected (e.g., cytochrome activity). Such information is important in evaluating of drug response and in investigation of possible causes of NMS.

## Conclusions

We have presented a case of the coincidence of WS and first-episode psychosis, which appeared during a COVID-19 lockdown. We hypothesized on the possible pathological relationships between the onset of the psychosis and severe anxiety in an individual with WS. This case report duly contributes to the limited literature on psychiatric comorbidity in WS.

## Data Availability Statement

The original contributions presented in the study are included in the article/supplementary material, further inquiries can be directed to the corresponding authors.

## Ethics Statement

Written informed consent was obtained from the individual(s) for the publication of any potentially identifiable images or data included in this article.

## Author Contributions

All authors listed have made a substantial, direct, and intellectual contribution to the work and approved it for publication.

## Conflict of Interest

The authors declare that the research was conducted in the absence of any commercial or financial relationships that could be construed as a potential conflict of interest.

## Publisher's Note

All claims expressed in this article are solely those of the authors and do not necessarily represent those of their affiliated organizations, or those of the publisher, the editors and the reviewers. Any product that may be evaluated in this article, or claim that may be made by its manufacturer, is not guaranteed or endorsed by the publisher.

## Abbreviations

WS: Williams syndrome
mU/L: milliunits per liter
CK: creatine kinase
NMS: neuroleptic malignant syndrome.

## References

[1] Morris CA. Williams Syndrome. In: Adam MP, Ardinger HH, Pagon RA, et al. editors. GeneReviews® [Internet]. Seattle, WA: University of Washington, Seattle 1993–2021 (1999) (accessed Mar 23, 2017). 20301427

[2] Pober BR Wang E Caprio S Petersen KF Brandt C Stanley T. High prevalence of diabetes and pre-diabetes in adults with Williams syndrome. Am J Med Genet C Semin Med Genet. (2010) 154C:291–8. 10.1002/ajmg.c.30261 20425788 PMC2882962

[3] Stinton C Tomlinson K Estes Z. Examining reports of mental health in adults with Williams syndrome. Res Dev Disabil. (2012) 33:144–52. 10.1016/j.ridd.2011.09.002 22093659

[4] Järvinen-Pasley A Bellugi U Reilly J Mills DL Galaburda A Reiss AL. Defining the social phenotype in Williams syndrome: a model for linking gene, the brain, and behavior. Dev Psychopathol. (2008) 20:1–35. 10.1017/S0954579408000011 18211726 PMC2892602

[5] Fisher MH Morin L. Addressing social skills deficits in adults with Williams syndrome. Res Dev Disabil. (2017) 71:77–87. 10.1016/j.ridd.2017.10.008 29032288

[6] Dodd HF Porter MA. Psychopathology in Williams syndrome: the effect of individual differences across the life span. J Ment Health Res Intellect Disabil. (2009). 2: 89–109. 10.1080/19315860902725867

[7] Valdes F Keary CJ Mullett JE Palumbo ML Waxler JL Pober BR. Brief report: major depressive disorder with psychotic features in Williams syndrome: a case series. J Autism Dev Disord. (2018) 48:947–52. 10.1007/s10803-017-3384-x 29164439

[8] Stinton C Elison S Howlin P. Mental health problems in adults with Williams syndrome. Am J Intellect Dev Disabil. (2010) 115:3–18. 10.1352/1944-7558-115.1.3 20025356

[9] Zarchi O Diamond A Weinberger R Abbott D Carmel M Frisch A. A comparative study of the neuropsychiatric and neurocognitive phenotype in two microdeletion syndromes: velocardiofacial (22q11.2 deletion) and Williams (7q11.23 deletion) syndromes. Eur Psychiatry. (2014) 29:203–10. 10.1016/j.eurpsy.2013.07.001 24054518

[10] Green T Gothelf D Glaser B Debbane M Frisch A Kotler M. Psychiatric disorders and intellectual functioning throughout development in velocardiofacial (22q11.2 deletion) syndrome. J Am Acad Child Adolesc Psychiatry. (2009) 48:1060–8. 10.1097/CHI.0b013e3181b76683 19797984

[11] Perälä J Suvisaari J Saarni SI Kuoppasalmi K Isometsä E Pirkola S. Lifetime prevalence of psychotic and bipolar I disorders in a general population. Arch Gen Psychiatry. (2007) 64:19–28. 10.1001/archpsyc.64.1.19 17199051

[12] Chang WC Wong CSM Chen EYH Lam LCW Chan WC Ng RMK. Lifetime prevalence and correlates of schizophrenia-spectrum, affective, and other non-affective psychotic disorders in the Chinese adult population. Schizophr Bull. (2017) 43:1280–90. 10.1093/schbul/sbx056 28586480 PMC5737409

[13] Savoja V Vicari S. Development of erosive gastrointestinal lesions during risperidone treatment in two patients with Williams syndrome. Prog Neuropsychopharmacol Biol Psychiatry. (2010) 34:711–2. 10.1016/j.pnpbp.2010.03.007 20227454

[14] Salgado H Martins-Correia L. Williams syndrome and psychosis: a case report. J Med Case Rep. (2014) 8:49. 10.1186/1752-1947-8-49 24520861 PMC3930303

[15] Thom RP Pober BR McDougle CJ. Psychopharmacology of Williams syndrome: safety, tolerability, and effectiveness. Expert Opin Drug Saf. (2021) 20:293–306. 10.1080/14740338.2021.1867535 33369485

[16] Braga RJ Reynolds GP Siris SG. Anxiety comorbidity in schizophrenia. Psychiatry Res. (2013) 210:1–7. 10.1016/j.psychres.2013.07.030 23932838

[17] Hartley S Barrowclough C Haddock G. Anxiety and depression in psychosis: a systematic review of associations with positive psychotic symptoms. Acta Psychiatr Scand. (2013) 128:327–46. 10.1111/acps.12080 23379898

[18] Cherniske EM Carpenter TO Klaiman C Young E Bregman J Insogna K. Multisystem study of 20 older adults with Williams syndrome. Am J Med Genet A. (2004) 131:255–64. 10.1002/ajmg.a.30400 15534874

[19] Järvinen A Korenberg JR Bellugi U. The social phenotype of Williams syndrome. Curr Opin Neurobiol. (2013) 23:414–22. 10.1016/j.conb.2012.12.006 23332975 PMC4326252

[20] Crespi BJ Procyshyn TL. Williams syndrome deletions and duplications: genetic windows to understanding anxiety, sociality, autism, and schizophrenia. Neurosci Biobehav Rev. (2017) 79:14–26. 10.1016/j.neubiorev.2017.05.004 28499504

[21] D Agostino A D'Angelo S Giordano B Cigognini AC Chirico ML Redaelli C. Brief psychotic disorder during the national lockdown in Italy: an emerging clinical phenomenon of the COVID-19 pandemic. Schizophr Bull. (2021) 47:15–22. 10.1093/schbul/sbaa112 32761196 PMC7454891

[22] Finatti F Pigato G Pavan C Toffanin T Favaro A. Psychosis in patients in COVID-19-related quarantine: a case series. Prim Care Companion CNS Disord. (2020) 22:20l02640. 10.4088/PCC.20l02640 32408399

[23] Shanbour A Khalid Z Fana M. Psychosis and infodemic isolation resulting in first inpatient hospitalization during the COVID-19 pandemic: a case series. Prim Care Companion CNS Disord. (2020) 22:20l02649. 10.4088/PCC.20l02649 32510878

[24] Tse L Barr AM Scarapicchia V Vila-Rodriguez F. Neuroleptic Malignant syndrome: a review from a clinically oriented perspective. Curr Neuropharmacol. (2015) 13:395–406. 10.2174/1570159X13999150424113345 26411967 PMC4812801

[25] Lee M Marshall D Saddichha S. Lurasidone-associated neuroleptic Malignant syndrome. J Clin Psychopharmacol. (2017) 37:639–40. 10.1097/JCP.0000000000000774 28806387

